# Human-Unrecognizable Differential Private Noised Image Generation Method

**DOI:** 10.3390/s24103166

**Published:** 2024-05-16

**Authors:** Hyeong-Geon Kim, Jinmyeong Shin, Yoon-Ho Choi

**Affiliations:** School of Computer Science and Engineering, Pusan National University, Busan 46241, Republic of Korea; qddd2000@pusan.ac.kr (H.-G.K.); sinryang@pusan.ac.kr (J.S.)

**Keywords:** data privacy, image de-identification, privacy-preserving deep learning

## Abstract

Differential privacy has emerged as a practical technique for privacy-preserving deep learning. However, recent studies on privacy attacks have demonstrated vulnerabilities in the existing differential privacy implementations for deep models. While encryption-based methods offer robust security, their computational overheads are often prohibitive. To address these challenges, we propose a novel differential privacy-based image generation method. Our approach employs two distinct noise types: one makes the image unrecognizable to humans, preserving privacy during transmission, while the other maintains features essential for machine learning analysis. This allows the deep learning service to provide accurate results, without compromising data privacy. We demonstrate the feasibility of our method on the CIFAR100 dataset, which offers a realistic complexity for evaluation.

## 1. Introduction

The rapid advancement in deep neural networks (DNNs) has enabled their widespread application in personalized services across diverse fields, including advertising [1], finance [2,3], and medicine [4,5,6]. To train these DNN models for such personalized services, institutions often collect and utilize extensive datasets. This data frequently contain sensitive information, raising concerns about user privacy and data protection.

The data memorization effect of DNNs, where models retain information beyond what is strictly necessary for their intended task [7], presents a significant privacy risk. This vulnerability enables malicious actors to target and extract sensitive data ‘memorized’ by the DNN. A prominent example is a model inversion attack [8,9,10], in which adversaries reconstruct representative input data from the DNN model itself, potentially exposing confidential information.

To mitigate sensitive information leakages, researchers have actively explored privacy-preserving deep learning (PPDL) techniques designed to maintain performance while protecting data. Two main categories have emerged: (1) encryption-based techniques, and (2) perturbation-based techniques.

Prominent encryption-based approaches include homomorphic encryption (HE) [11,12,13,14,15] and secure multi-party computation (SMPC) [16,17,18]. These methods encrypt DNN computations and values, ensuring the data remain unintelligible to unauthorized users without the necessary decryption keys. However, the substantial computational overheads introduced by these techniques often make them impractical. Additionally, the complex service architectures, frequently involving trusted third parties, can introduce vulnerabilities to privacy attacks [19].

In contrast to encryption-based techniques, perturbation-based methods modify DNN models or input data to prevent the reconstruction of the original information. Differential privacy (DP) [20] has emerged as a widely adopted model modification technique, due to its low computational overheads. A prominent example is differential private stochastic gradient descent (DP-SGD) [21], which introduces DP noise during the DNN training process. However, An et al. demonstrated that DP-SGD remains vulnerable to model inversion attacks, especially with clean, raw input data and a known input domain [22].

Input data modification strategies, such as marginal distribution (MD)-based techniques [23,24], offer an alternative. While effective for structured tabular data, their application is limited in this domain. Generative adversarial networks (GANs) [25,26,27] can address both structured and unstructured (e.g., image) data, but their success has been primarily confined to simple datasets like MNIST. Instance-hiding based methods [28] have shown good performance for accuracy on various datasets. However, they can be applied in the training phase only. Thus, such methods cannot be applied to protect a client’s data privacy.

Inspired by a recent study demonstrating that machines can achieve higher recognition rates on strategically noised images [29], we propose a differential privacy-based image generation method for image datasets. Our approach aims to make images unrecognizable to humans, preserving privacy, while enhancing machine-recognizability for specific tasks. We address two core questions: (1) how to augment machine-interpretable characteristics within the noised images, and (2) how to disrupt the original image’s explanatory cues, mitigating privacy vulnerabilities. To enhance machine-readability, we leverage explainable artificial intelligence (XAI) techniques. By extracting pretrained features from machine models and strategically embedding them into the noised images, we ensure that they remain interpretable by subsequent machine learning systems.

Main contributions of this paper can be summarized as follows:

1. We propose an image de-identification method that strategically combines two types of noise. The first type makes the image unrecognizable to humans, protecting privacy. The second type preserves essential features for machine learning tasks. This dual-noise approach effectively removes private information from input images, while maintaining the data’s utility for downstream analysis.
2. Our proposed method enables privacy-preserving deep learning-based services, by de-identifying client input images, protecting their data during service interactions.
3. Our experimental results demonstrate the potential of XAI techniques for generating features or clues that facilitate DNN classification. We anticipate that these findings will stimulate future research directions focused on enhancing privacy across diverse data modalities.

The rest of this paper is organized as follows. In Section 2, we describe related works. We propose our differential private image de-identification method and give a theoretical analysis in Section 3 and Section 4, respectively. In Section 5, we show the feasibility of the proposed method with experimental results. Finally, we summarize this paper in Section 6.

## 2. Related Works

### 2.1. Perturbation-Based Privacy-Preserving Deep Learning

Differential private stochastic gradient descent (DP-SGD) [21] stands as a prominent perturbation-based PPDL method. It protects privacy by injecting calibrated noise into the model’s gradients during the training process. This allows the model to learn essential features, while adhering to the rigorous mathematical guarantees of differential privacy, which quantify privacy protection based on the chosen parameters. However, recent advancements in model inversion attacks have demonstrated that DP-SGD remains vulnerable, even with carefully tuned privacy settings [22].

In contrast to model modification methods, input modification approaches probabilistically alter sensitive information in the original data, hindering malicious exploitation of DNN models. Two notable examples include DataSynthesizer by Ping et al. and the Bayesian network method proposed by Zhang et al. [23,24]. DataSynthesizer offers differential privacy by analyzing dataset distributions and feature correlations, while Zhang et al. employed a differential private Bayesian network for data synthesis. However, a key limitation of both methods is their reliance on Bayesian theory, making them primarily suitable for structured tabular data, where data distributions are well-defined.

GAN-based approaches offer an alternative for handling unstructured data, where data distributions are less well defined. Notable examples include DP-GAN by Xie et al. [25], which incorporates DP-SGD into the GAN’s generator, and the teacher–student GAN model proposed by Jordan et al. [26] for differential private generation. Torkzadehmahani et al. [27] further extended DP-GAN with a conditional framework for label generation. However, a significant limitation remains: these methods have primarily demonstrated feasibility on simple datasets, either structured or like MNIST.

**Figure 1:**
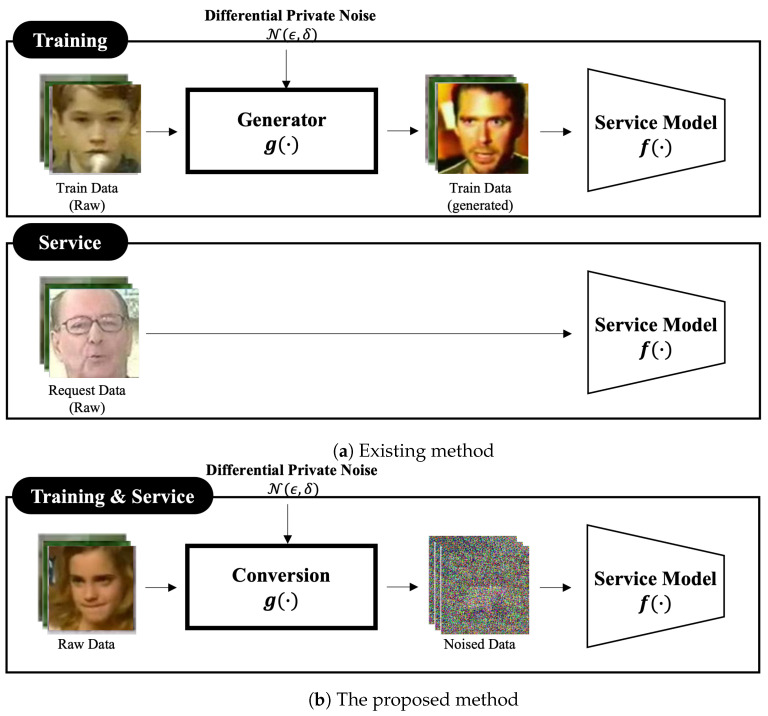
Architecture comparison of the existing and the proposed input modification schemes.

Huang et al. introduced instance-hiding as an alternative input modification approach [28]. Their method strategically blends pixels from private data with those from a large, diverse public dataset. This obfuscates the original content, while selectively preserving key features, enabling machine learning on the modified data and subsequent classification of clean inputs. However, a critical vulnerability remains: during service, the instance-hiding method receives clean, raw data, exposing its distribution and leaving it susceptible to state-of-the-art model inversion attacks. Furthermore, as illustrated in Figure 1a, this lack of service-phase protection prevents clients from securely submitting their private data for analysis by the service model.

To address the service-phase vulnerability of instance-hiding, Gao et al. proposed an image obfuscation method specifically for medical images [18]. Their method randomizes both pixel values and positions within a defined distribution, achieving a balance between practicality and client-side applicability. However, this randomization approach presents a significant limitation: it disrupts the spatial relationships between pixels, making the method unsuitable for services where object location is crucial, such as object-detection tasks.

### 2.2. Model Inversion Attack

Since the seminal work on model inversion attacks in [8], the field has witnessed the rapid development of increasingly sophisticated techniques. In this section, we survey recent advancements in state-of-the-art model inversion attack research.

Balle et al. introduced a black-box attack capable of reconstructing training data [30]. Assuming knowledge of the data distribution, they leveraged shadow models and confidence scores from the target model. Wang et al. proposed a method combining variational autoencoders with StyleGAN [31,32]. By training a prior distribution for the latent space that reflects the training data distribution, they could generate representative latent vectors suitable for StyleGAN. An et al. also employed StyleGAN, but with a different approach [22]. They trained a latent space mapping network using the target classifier’s confidence scores. This allowed them to extract representative data of the target class, bypassing the defenses of DP-SGD-based PPDL models, even with strong privacy settings.

The current landscape of model inversion attacks is dominated by black-box approaches. These attacks require minimal information—only the target model and the data distribution—posing a significant challenge to privacy-preserving deep learning. Since DP-based PPDL methods inherently reveal the data distribution, they are particularly susceptible to such attacks. This highlights the critical need for further research into developing robust privacy-preserving techniques that can withstand these increasingly sophisticated black-box attacks.

### 2.3. Explainable Artificial Intelligent

The widespread adoption of machine learning (ML) has spurred questions about whether models arrive at their classifications using features that align with human understanding. To address this, explainable artificial intelligence (XAI) has emerged as a field dedicated to developing techniques that reveal the features influencing ML model outputs. XAI aims to provide transparency and insights into the decision-making processes of these complex models.

Several XAI techniques exist for explaining DNN behavior. Gradient-based methods, such as those proposed by Simonyan et al. [33], utilize backpropagation gradients to generate heatmap-style explanation maps. Guided backpropagation [34] builds upon this concept but drops negative gradients, aiming to highlight only the positive contributions of input features to the final output. Layer-wise relevance propagation (LRP) [35,36] takes a different approach, analyzing the activated weights within the DNN. By calculating the contribution of each weight, LRP generates an explanation map that reveals how individual features influenced the model’s decision.

## 3. Human-Unrecognizable Differential Private Noised Image Generation Method

Existing input modification-based PPDL schemes often utilize unmodified original images during the service time, as shown in Figure 1a. This exposes client data to potential privacy risks, including network hijacking. While encryption offers a degree of protection, it has known vulnerabilities to certain privacy attacks [19]. Alternatively, adding substantial noise to the data can provide robust privacy by completely obscuring sensitive information. However, this extreme obfuscation makes the image unusable for both authorized humans and machine learning models, negating its utility.

To address the limitations of traditional privacy-preserving methods, we propose a noise generation method that makes images unrecognizable to humans, while preserving machine-readability. Our approach combines two core elements:

1. **Human Obfuscation:** We introduce strong Gaussian noise into the image, disrupting the visual coherence and making it unrecognizable to humans.
2. **Machine-Readable Enhancement:** Leveraging XAI techniques, we extract crucial image features, such as structural information, and re-embed them into the noised image. This maintains machine-recognizability, despite the obfuscation.

This hybrid method effectively safeguards privacy, while enabling machine learning analysis.

Recent research on DNN security, particularly in adversarial examples and XAI, provides a compelling foundation for our approach. These studies highlight a critical disparity: features readily identifiable by humans may not be the same features crucial for machine recognition [37,38]. For instance, an image and its adversarial counterpart can appear identical to humans, yet be classified differently by a machine learning model. Similarly, Dombrowski et al. demonstrated that explanation maps, tools used for understanding DNN decisions, can diverge for visually indistinguishable images [38]. By leveraging this inherent disparity, our method injects machine-recognizable features into images, achieving human-imperceptible obfuscation, while preserving machine readability.

### 3.1. Overview

Figure 1b shows a simplified architecture of the proposed differential private image generation method. The proposed method consists of two components; i.e., *Conversion* g(·) and *Service Model* f(·). The *Conversion* component transforms the input image into a noised format, preserving machine-readability, while obscuring the image from human recognition, and the *Service Model* component conducts training and analysis tasks on the converted, noised image.

Our two-component system, consisting of a data de-identification module and an analysis service, offers a promising approach for securing client privacy during data transfer. This architecture leverages DP and is divided into three phases:

**Phase 1: Training with Privacy Protection**

1. **Distribution Learning:** The de-identification module, denoted as g(·), learns the distribution of Gaussian noise and the machine-readable features essential for accurate analysis. XAI techniques are employed to extract these features. However, it is crucial to acknowledge that such features might introduce some level of information leakage.
2. **Privacy-Preserving Noise Injection:** To mitigate information leakage risks arising from a potential malicious analysis of the service model (f(·)), DP noise is incorporated into the training process of g(·). This added noise mathematically guarantees a level of privacy protection against leakage through the service model.
3. Service Model Training: Once g(·) has been trained, the noisy images generated by g(·) are used to train the service model f(·).

**Phase 2: Secure Distribution of De-identification Module**

The trained g(·) component is then securely delivered to authorized clients through a secure channel.

**Phase 3: Client-Side Data Conversion and Secure Analysis**

1. **Client-Side Conversion:** When a client has private data containing sensitive information to be analyzed, the client utilizes the locally deployed g(·) component. This component transforms the client’s data into a machine-readable but human-imperceptible noisy image.
2. **Secure Transmission and Analysis:** The anonymized noisy image is then transmitted to the service provider. The service model f(·), previously trained on similar noisy images, can perform the required analysis, without compromising the client’s raw data privacy, due to the DP guarantees and human-imperceptibility of the noise.

### 3.2. Conversion Component

This section delves into our core component, termed the conversion component (g(·)), which addresses two crucial questions in privacy-preserving deep learning: (1) How can images be obfuscated for privacy, while retaining features essential for machine learning?; and (2) How can we eliminate potential information leakages through explanation maps that arise from obfuscated images?

To move beyond the limitations of purely random Gaussian noise, we replace it with a generator that learns to produce noise with a controlled distribution. This balances obfuscation with structure preservation. Furthermore, we employ XAI techniques to extract machine-readable features. Unlike traditional CNN feature layers or pretrained teacher models, which are strongly tied to specific details within the original image, XAI results have a weaker connection to the image itself. XAI results instead highlight how the model reached its output, similar to the backpropagation process. This characteristic makes them ideal for extracting machine-readable features without excessively compromising privacy.

A significant challenge lies in the potential for an image’s partial shape or features to persist within its XAI-generated explanation map, even after obfuscation. To combat this, we add a controlled degree of randomness to the noisy image’s explanation map during the training process of our conversion component. However, indiscriminate random perturbations risk damaging the very features we aim to preserve for analysis. Instead, we carefully overlay the explanation map with that of a different, randomly chosen class. This process is meticulously managed using DP, ensuring mathematical guarantees regarding the privacy level offered.

**Figure 2:**
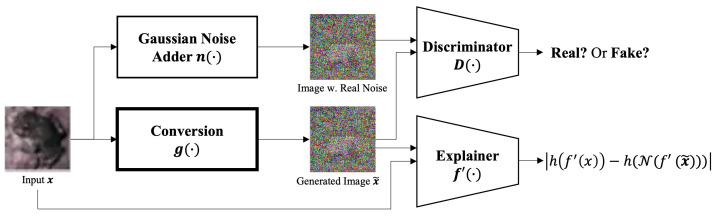
GAN architecture for the proposed image conversion component.

To train our conversion component (g(·)), we employ a specialized generative adversarial network (GAN). This GAN incorporates a discriminator that guides the generator towards producing structured, target-magnitude Gaussian noise, and an explainer ($f^{\prime }\left(\cdot \right)$) that facilitates the generation of machine-readable features using XAI-derived explanation maps (see Figure 2). The explainer’s architecture mirrors that of the service model (f(·)), and it is pretrained with the original, unmodified data. For the given explainer XAI method h(·), Gaussian noise generation method n(·), differential private noise generation method N(·), and specified input image *x*, the objective function of our GAN algorithm can be expressed as

(1) $$\begin{array}{cc} \min_{g}\max_{D}\; & E_{n\left(x\right)∼p_{\mathrm{data}}\left(n\left(x\right)\right)}\left[\log D(n(x)))\right]\\ \; & +E_{x∼p_{\mathrm{data}}\left(x\right)}[1-\log D(g(x))\\ \; & +|h\;\circ \;f^{\prime }\left(x\right)-h\circ N\circ f^{\prime }\left(g\left(x\right)\right)\left|\right] \end{array}$$

where ∘ is the composition of functions, g(·) is a generator, which is the *Conversion* component, and *D* is a discriminator. In Equation (1), the term $|h\;\circ \;f^{\prime }\left(x\right)-h\circ N\circ f^{\prime }\left(g\left(x\right)\right)|$ means the distance between the explain map of the original data and the explain map of the converted data with differential private noise. Here, the loss function of the discriminator is the same as that of the original GAN’s discriminator, except that the distribution of the discriminator’s input is changed from the original images to noised images. Contrarily, the loss function of the generator is defined as a joint loss consisting of noise loss and explanation loss.

The noise loss $L_{noise}$ measures the errors of the discriminator for modified image data G(x) and is calculated as follows:

(2) $$\begin{array}{c} L_{noise}=\log\left(1-D(g(x))\right) \end{array}$$

which encourages the *Conversion* component to learn the distribution of Gaussian noise.

The explanation loss $L_{exp}$ measures the difference between the explain maps of the original image and the modified image and is calculated as follows:

(3) $$\begin{array}{c} L_{exp}=d\left(h\circ f^{\prime }\left(x\right),h\circ N\circ f^{\prime }\left(g\left(x\right)\right)\right) \end{array}$$

where d(·) is a distance function that measures the difference between the explain maps of the original image $h\circ f^{\prime }\left(x\right)$ and the converted data with differential private noise $h\circ N\circ f^{\prime }\left(g\left(x\right)\right)$. The explanation loss makes features of the original image and the converted image more similar when analyzed by machine; that is, it encourages the *Conversion* component to inject machine-recognizable features into the converted image.

Consequently, the loss function of the generator $L_{G}$ is defined as a weighted summation of two such loss functions:

(4) $$\begin{array}{c} L_{g}=\alpha L_{noise}+\beta L_{exp} \end{array}$$

where α and β are hyperparameters to control the characteristics of the generator.

As mentioned above, the role of the discriminator is to encourage the *Conversion* component to learn the distribution of Gaussian noise. In other words, the distribution of the input images is changed to the distribution of noised images. Thus, the loss function of the discriminator $L_{D}$ is defined as follows:

(5) $$\begin{array}{c} L_{D}=\log\left(1-D(n(x))\right)+\log\left(D(g(x))\right) \end{array}$$

## 4. Theoretical Analysis

To show that the security of the proposed method can be supported by DP, we describe our theoretical basis in this section. According to the definition of DP, the proposed method has to satisfy following equation:

(6) $$\begin{array}{c} \frac{Pr[f\left(D_{1}\right)=S]}{Pr[f\left(D_{2}\right)=S]}\leq e^{\epsilon }+\delta \end{array}$$

where f(·) is a deterministic function that hides a single data point. $D_{1}$ and $D_{2}$ are neighboring data points in a specific data distribution. ϵ and δ are privacy parameters. The distinguishability of two processed data points, $f(D_{1})$ and $f(D_{2})$, increases when the value of ϵ and δ increases.

In Equation (6), to address neighboring data $D_{1}$ and $D_{2}$, we need to define the field of data. Since the proposed method is focusing on image data, the field of data can be *width* · *height* · *channels* · 255 in RGB expression. However, such a field only represents the field of each pixel, not the contents in the image. Since the content in the image is expressed by a group of pixels, we define a set of classification results as a field of data.

Therefore, the conversion component of the proposed method can be expressed as follows:

(7) $$\begin{array}{c} h\circ N\circ f^{\prime }\left(g\left(x\right)\right) \end{array}$$

where N is a deterministic function, which generates noise satisfying DP, which is equivalent to *f* in Equation (6). Here, we apply a theorem that defines the properties of DP as follow [39]:

**Theorem** **1.** *If F(x) satisfies DP, then for any deterministic or randomized operation g on F(x), g(F(x)) satisfies DP.*

Therefore, following the Theorem 1, the conversion component satisfies DP.

## 5. Experiment

To show the feasibility of our proposed scheme, we show experimental results as follows: (1) the classification performance according to the values of noise magnitude σ and privacy parameter ϵ and δ; (2) a de-identification performance comparison of the image with the original Gaussian noise and the image with the proposed scheme; (3) an analysis on machine-recognizable features from XAI; and (4) the effect of DP on the explanation map.

### 5.1. Experimental Configuration

Since most reference implementations of XAI do not support gradient calculation from explanation maps, we implemented an explainer network that calculates gradients from explanation maps referring to Dombrowski et al.’s implementation [38]. The *Conversion* component was implemented with a generator, as shown in Table 1, and a *Service Model* component that performed classification on the generated images, the VGG16 network shown in Table 2 with input size of 224 × 224 pixels, was used.

In addition, we performed all experiments using the CIFAR100 [40] dataset. Existing DP-based data-modification-based PPDL approaches, such as [25,27], were used MNIST as a benchmark dataset. However, since the MNIST dataset has a small number of classes and too simple a structure, the MNIST dataset was not suitable to show the possibility of general applications to various fields. On the other hand, the CIFAR100 dataset consists of very diverse types of images divided into 100 classes. Due to the diversity of the composed images, it is used to show the general applicability of new techniques in state-of-the-art research related to image processing [41,42,43,44]. Therefore, to show the feasibility of the proposed human-unrecognizable differential private noised image generation method, we used the CIFAR100 dataset in the experiments.

The differential private noise generation mechanism and the XAI method for the explainer were set up as a Gaussian mechanism [45] and guided backpropagation [34], respectively.

### 5.2. Classification Performance

To show the feasibility of the proposed human-unrecognizable differential private noised image generation method, we evaluated the classification accuracy on the CIFAR100 dataset in two ways: (1) measurement of the classification accuracy under various parameters; and an (2) ablation test without $L_{exp}$. Here, the accuracy of the explainer model used to train the *Conversion* component that satisfied the parameters was 0.720. The overall results are shown in Table 3.

First, we measured the accuracy of the *Service Model* component trained with the generated noised training images using the *Conversion* component. Each *Conversion* component was trained with noise parameter σ values of 3.0, 4.0, and 5.0 and privacy parameters (ϵ,δ) value (0.1, $10^{-5}$), (0.1, 0.9), (0.9, $10^{-5}$) and (0.9, 0.9), respectively. When the σ value was 3.0 and ϵ value was 1, the accuracy was observed to be about 0.62 with both of δ values. Similarly, with a σ value of 4.0, the accuracy was about 0.61 and 0.63 with ϵ values of 0.1 and 0.9, respectively. For a σ value of 5.0, accuracy was about 0.60 and 0.62 with ϵ values of 0.1 and 0.9, respectively. From such results, we observed a tendency that matched with the theoretical characteristic of DP, where the value of ϵ affected the noise more than that of δ. In the ablation test, we observed accuracies of 0.603, 0.586, and 0.577 with respect to σ values of 3.0, 4.0, and 5.0, respectively. Such results imply that the explanation loss $L_{exp}$ is a key feature for injecting machine-recognizable features into a converted image. Considering the simple network architecture of the *Service Model* component and the complexity of the CIFAR100 dataset, such results were considered acceptable [46].

The *Conversion* component of the proposed scheme mimics the Gaussian noise and differential private explainer’s explanation maps; that is, it is infeasible to show the privacy of modified images with mathematical theory. Instead, we show that the noise generated from the *Conversion* component is similar enough to the noised image with real Gaussian noise.

### 5.3. Image De-Identification

**Figure 3:**
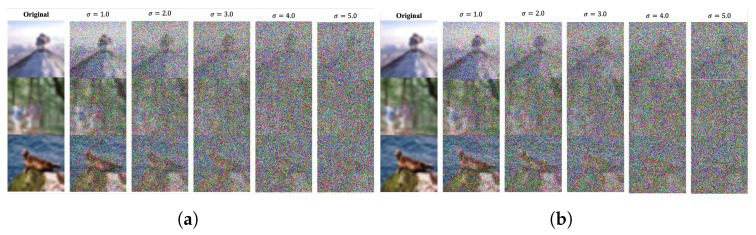
Comparison of noised images with Gaussian noise and converted images using the proposed scheme. (**a**) Noised images with Gaussian noise with a target σ value; (**b**) Converted images using the proposed scheme with a target σ value (ϵ=0.1,δ=0.9).

Figure 3 shows graphical examples of images with the original Gaussian noise and converted images using the proposed scheme. According to the characteristic of Gaussian noise, the magnitude of noise increases when the σ value increases. In other words, details of the original image fade out and the probability of a sensitive information leakage through human analysis decreases, Figure 3a. Specifically, it is very difficult to recognize the presence of any object in the image with a value of σ around 3.0 and more. Images converted by the proposed *Conversion* component under each target σ are shown in Figure 3b. When comparing the images with Gaussian noise and converted images, it is almost impossible to see a distinctive difference.

To show the de-identification performance concretely, we measured a quantitative indicator of Structural Similarity (SSIM) [47], which is widely used in the image processing field to measure a generated image’s quality. The SSIM quantifies the perceived similarity between two images, often referred to as the reference image (*x*) and the test image (*y*). It goes beyond a simple pixel intensity comparison by incorporating luminance (l(x,y)), contrast (c(x,y)), and structure (s(x,y)) comparisons. The SSIM value ranges from −1 to 1, with 1 indicating perfect structural similarity and values closer to −1 signifying significant structural dissimilarities. The specific equation for SSIM involves a combination of these three components:

(8) SSIM(x,y)=[l(x,y)×c(x,y)×s(x,y)]

where each component is calculated based on the means ($\mu_{x}$, $\mu_{y}$), standard deviations ($\sigma_{x}$, $\sigma_{y}$), and covariance ($\sigma_{xy}$) of *x* and *y* within a local window, along with two parameters (C1 and C2) to stabilize the division by small denominators. This metric provides a valuable tool for assessing image quality and compression effectiveness in image processing applications. Therefore, we calculated the SSIM between the original image and the modified image, to show the average difference from the original image. In other words, a smaller SSIM value means a lower recognition probability by humans. Table 4 shows the average SSIM of the test datasets of CIFAR10 and CIFAR100, which were generated with the original Gaussian noise and the proposed scheme. In both datasets, the average SSIM value decreased as the target σ increased. In addition, we observed that there was no difference in the average SSIM of noised images and converted images according to each target σ and privacy parameter ϵ. That is, the proposed *conversion* component showed a very stable de-identification performance.

### 5.4. Machine-Recognizable Features from XAI

**Figure 4:**
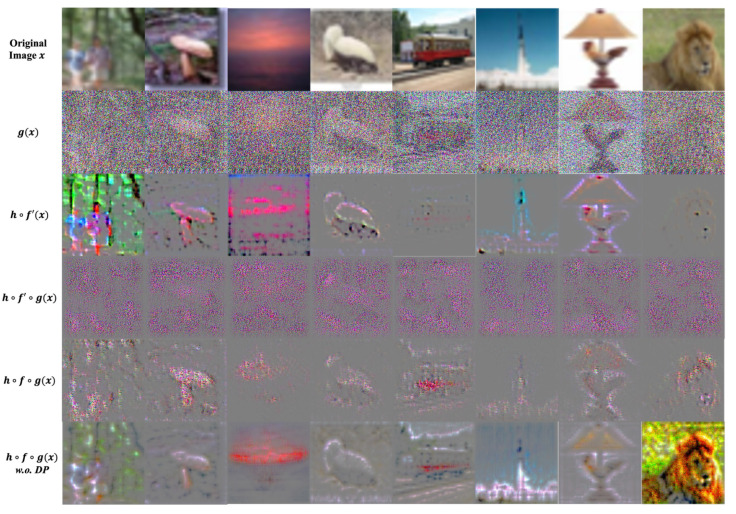
Comparison of the explanation map extracted from the explainer and the service model ($\sigma =3.0,\epsilon =0.1,\delta =10^{-5}$).

To analyze the feasibility of the proposed machine-recognizable feature injection method, we visualized some features analyzed by machine using XAI, i.e., guided backpropagation, and Figure 4 shows the results. The first row shows the original image of samples, and the corresponding converted image by *Conversion* component g(x) is shown in the second row. In the third row, an explanation map of the classification results of the explainer model of the original image $h\circ f^{\prime }\left(x\right)$ is shown. Explanation maps of the classification results of the explainer model $h\circ f^{\prime }\circ g\left(x\right)$ and the *Service Model* component h∘f∘g(x) are shown in the fourth and fifth row, respectively. The last row shows explanation maps of the classification results of the model where DP was not applied. All samples were selected from the test set of the CIFAR100 dataset, which was classified correctly by the *Service Model* trained with σ=3.0, ϵ=0.1 and $\delta =10^{-5}$.

Considering Equation (3), the explanation map of $h\circ f^{\prime }\left(x\right)$ and $h\circ f^{\prime }\circ g\left(x\right)$ should have become similar as the model shows better performance. However, the explanation maps of $h\circ f^{\prime }\circ g\left(x\right)$ in the fourth row only shows very strange images that do not match with the corresponding explanation map in the third row. In addition, the explanation map of $h\circ f^{\prime }\left(x\right)$ and h∘f∘g(x) show different shapes with proper privacy parameter settings. However, the experimental results in Section 5.2 show a clear performance difference using $L_{exp}$. Additionally, compared to the last row, which shows almost all details, the explanation maps in the fifth row hide details of the object very well. As a consequence of such observations, we conjecture that the explanation loss $L_{exp}$ preserves machine-recognizable features that are not human-unrecognizable, even in the presence of noise from DP.

### 5.5. Effect of DP on the Explanation Map

**Figure 5:**
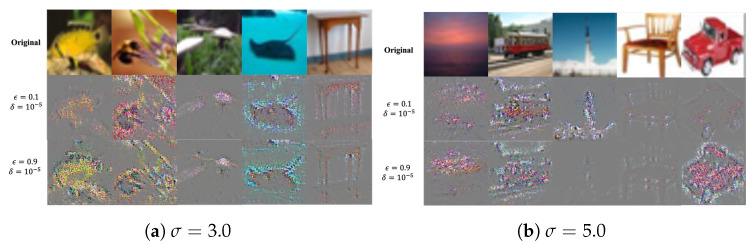
Comparison of noised images with Gaussian noise and converted images using the proposed method.

To observe the effect of DP on the converted image, we extracted an explanation map from arbitrary selected samples under various privacy parameters. All samples were selected from the test set of the CIFAR100 dataset that was classified correctly by all *Service Models* with each privacy parameter. Figure 5 shows the partial results of the selected samples. In each sub-figure, the first row shows the original image of each sample; the second row shows the corresponding explanation map with a stronger privacy parameter; and the third row shows another corresponding explanation map with weaker privacy parameters.

As shown in Figure 5a, most explanation maps show the outlines of the object with a relatively low target σ value. However, when considering privacy parameters, we can observe that explanation maps with weaker parameters show colors and shapes similar to those of the original object. In particular, in the first sample from left, the shape of the fish has almost disappeared in the second row. Conversely, the third row shows a much clearer shape and colors of the yellow fish. Meanwhile, with a relatively high target σ value, the explanation maps show very noisy images, regardless of the privacy parameters. However, we can intermittently observe relatively clear explanation maps, such as the last image of the third row from the left.

## 6. Conclusions

The proposed method presents a new privacy-preserving deep learning method that tackles the challenge of protecting client data during service interactions. Our contribution lies in differential privacy-based image de-identification. This method strategically injects noise to obfuscate visual content, while strategically embedding machine-readable, XAI-derived features. We achieve this balance using a customized GAN architecture that explicitly incorporates explanation maps during the training process.

In addition, our approach addresses the limitations of the existing perturbation-based methods, which can be vulnerable to state-of-the-art model inversion attacks. The integration of differential privacy (DP) provides theoretical guarantees of privacy, with the level of protection controlled by the DP parameters.

The optimization of the accuracy and privacy trade-offs caused by image de-identification, as well as differential privacy and extensions to other types of tasks, such as object detection, will be our future work.

## Figures and Tables

**Table 1:** Details of the implemented generator network for the conversion component.

Layer	Description	Number of Parameters
Convolution (7 × 7, stride 1, padding 3)	64 filters	1792
Instance Norm	-	-
ReLU activation	-	-
(6 residual blocks)	…	…
Deconvolution (3 × 3, stride 1, padding 1)	64 filters	4352
Instance Norm	-	-
ReLU activation	-	-
Convolution (7 × 7, stride 1, padding 3)	Target image channels	1792
tanh activation	-	-

**Table 2:** Details of the implemented VGG16 network.

Layer	Description	Number of Parameters
Convolution (3 × 3 , same padding)	64 filters, ReLU activation	1792
Convolution (3 × 3, same padding)	64 filters, ReLU activation	36,864
Max Pooling (2 × 2, stride 2)	-	
Convolution (3 × 3, same padding)	128 filters, ReLU activation	73,792
Convolution (3 × 3, same padding)	128 filters, ReLU activation	147,520
Max Pooling (2 × 2, stride 2)	-	
Convolution (3 × 3, same padding)	256 filters, ReLU activation	295,136
Convolution (3 × 3, same padding)	256 filters, ReLU activation	590,080
Convolution (1 × 1)	256 filters, ReLU activation	65,536
Convolution (3 × 3, same padding)	256 filters, ReLU activation	590,080
Max Pooling (2 × 2, stride 2)	-	
Fully-connected	4096 units, ReLU activation	1,048,576
Fully-connected	4096 units, ReLU activation	16,777,216
Output (Softmax)	100 units	40,960

**Table 3:** Classification accuracy of the proposed method under various parameter settings (w. base accuracy 0.720).

Privacy Parameter		Noise Parameter		
		σ=3.0	σ=4.0	σ=5.0
without $L_{exp}$		0.603	0.586	0.577
ϵ=0.1	$\delta =10^{-5}$	0.620	0.611	0.601
	δ=0.9	0.623	0.612	0.598
ϵ=0.9	$\delta =10^{-5}$	0.652	0.629	0.624
	δ=0.9	0.651	0.632	0.620

**Table 4:** Average SSIM of images where the original Gaussian noise and the proposed image conversion method were applied (δ=0.9, scaled by $\times 10^{2}$).

Scheme	Gaussian			Proposed								
				ϵ=0.9			ϵ=0.5			ϵ=0.1		
**Target** σ	3.0	4.0	5.0	3.0	4.0	5.0	3.0	4.0	5.0	3.0	4.0	5.0
**SSIM**	1.54	1.29	1.16	1.52	1.21	1.12	1.54	1.30	1.15	1.55	1.28	1.22

## Data Availability

The all data including source codes for implementation is available on https://github.com/sinryang/You-Know-Nothing, accessed on 10 March 2024.
